# Advances in the Experimentation and Numerical Modeling of Material Joining Processes (Second Edition)

**DOI:** 10.3390/ma18184235

**Published:** 2025-09-09

**Authors:** Raul D. S. G. Campilho

**Affiliations:** 1 CIDEM, ISEP—School of Engineering, Polytechnic of Porto, R. Dr. António Bernardino de Almeida, 431, 4200-072 Porto, Portugal; raulcampilho@gmail.com; 2 INEGI—Institute of Science and Innovation in Mechanical and Industrial Engineering, Pólo FEUP, Rua Dr. Roberto Frias, 400, 4200-465 Porto, Portugal

Material joining processes are fundamental in modern structural engineering, by combining components into functional assemblies that meet the performance, safety, and economic requirements of the industry [1,2]. In recent years, the design and manufacturing of load-bearing structures have been driven by new trends in materials development, sustainability considerations, and multi-material solutions [3]. However, these developments pose challenges for engineers, particularly in the selection and optimization of joining techniques [4,5,6]. Differences in melting points, thermal expansion coefficients, and stiffness, coupled with the need to join thin-walled or dissimilar materials, necessitate that the mechanical performance, durability, manufacturability, and cost-effectiveness of the joined structures are duly considered in the design stages [7]. In parallel to traditional processes such as welding, brazing, riveting, and mechanical fastening, the industry has also adopted advanced and hybrid methods, including adhesive bonding, clinching, friction stir welding, laser welding, and diffusion welding, each with its own strengths and limitations [8,9,10,11]. The evolution of these techniques was triggered by performance demands and also requirements for greener manufacturing, process efficiency, and the integration of quality assurance during the design process [12]. Experimental investigations remain indispensable to validate numerical approaches, but numerical modeling has become a powerful tool, able to simulate complex geometries, loading scenarios, and the behavior of advanced materials, including those exhibiting plasticity, anisotropy, or time-dependent degradation [13]. Despite their scientifically proven potential, numerical approaches such as the finite element method and fracture mechanics modeling are still underutilized in industrial practice, especially for applications involving high strain rates, fatigue, or impact loading. Thus, it is essential to reduce distances between academic research and industrial application, by developing robust models validated by comprehensive experimental data [14,15,16]. This Special Issue, entitled “Advances in the Experimentation and Numerical Modeling of Material Joining Processes (Second Edition)”, was conceived to disseminate the latest progress in these areas, by gathering high-quality contributions that advance both the understanding and application of joining processes. The twelve contributions present in this Special Issue are divided into four thematic areas, described next with short descriptions of selected contributions from each area.

**Adhesive Bonding: Fundamentals, Environment Effects, and Fatigue Performance**

Adhesive bonding is nowadays widely used in engineering applications, mostly due to lightweight and multi-material structure requirements by the industry [17]. Unlike welding or mechanical fastening, adhesives enable uniform stress distribution, eliminate the need for holes or heat input, and allow the joining of dissimilar substrates [18,19]. Nonetheless, despite these advantages, adhesive joints face some limitations: curing control, limited shelf lives, defect formation, rigorous surface preparation, and ageing in aggressive environments [20,21]. Addressing these issues requires advances in material formulations and systematic studies on durability, defect mechanisms, and predictive modeling frameworks [22,23,24]. This Special Issue includes a strong contribution to understanding, improving, and ensuring the long-term reliability of adhesive joints under varying conditions. The review paper of Dachev et al. [25] on adhesive bonding covers the mechanical and molecular adhesion mechanisms in these joints, common adhesive types, and adhesive bond applications. It discusses how defects, environmental ageing, and inadequate surface preparation can negatively affect joint integrity, while stressing the importance of proper bond line design. The study further underscores the importance of moving toward sustainable bonding solutions, emphasizing practices that allow for recycling and straightforward disassembly, particularly relevant in sectors such as aerospace and automotive. In addition, the authors point out the current absence of a comprehensive framework for evaluating defects and their interactions. They argue that progress in this area will require the integration of advanced modeling techniques, complementary inspection methods, and the development of bio-based adhesives to ensure greater reliability and to support the transition toward a circular materials economy. Abenojar et al. [26] explore how the type of metallic substrate affects the curing process of high-strength anaerobic adhesives used in structural joints. By using differential scanning calorimetry (DSC), the authors estimate the activation energy and other kinetic parameters for bonds on copper and iron. Results show that copper substrates promote faster and more complete curing by lowering the activation energy, enhancing overall adhesive performance. The work provides practical principles to adjust adhesive formulations to specific metals in industrial applications.

**Structural Optimization and Reinforcement Strategies for Bonded Joints**

Apart from adhesive formulation, the geometry of the joint itself plays a decisive role in performance [27,28]. Traditional designs are often associated with stress concentrations that initiate early failure, while in high-performance sectors such as aerospace, additional reinforcement is sometimes required to ensure damage tolerance [29]. The geometric design of adherends and the integration of reinforcement elements can significantly increase the strength, stiffness, and damage tolerance of bonded joints [30,31]. An example of reinforcement is using through-thickness pins to provide extra resistance against delamination and disbond growth [32]. The surge of additive manufacturing (AM) has opened new design possibilities, making it possible to tailor the adherend geometries with optimized topologies that distribute stresses more evenly across the adhesive layer [33]. Two contributions in this Special Issue show how innovation in joint architecture can provide performance gains. Ascher and Späth [34] demonstrate how additive manufacturing design freedom can be used to enhance adhesive joint performance in hybrid structures. A carbon fiber composite tube was joined to a powder-bed-fusion aluminum sleeve, whose topology was optimized via finite element analysis to achieve more uniform adhesive stress distribution. The optimized design reduced stress peaks by 75% without affecting static strength but significantly extended fatigue life. Bhagatji et al. [35] evaluate the effect of post-cured through-thickness reinforcement (PTTR) on the crack resistance of skin-stringer composite assemblies. Tests on pristine and pre-disbonded specimens showed strength gains of 15.5% and 20.9%, respectively, with finite element pin-spring modeling being able to effectively replicate the stiffness, adhesive strength, and progressive pin failure.

**Welding and Surface Processing: Experimental Analysis and Predictive Models**

Despite the increasing research on hybrid and adhesive-based techniques, welding remains indispensable in structural engineering, particularly for metals and high-temperature alloys [36,37]. Rotary friction welding and related methods are gaining attention because they minimize defects and residual stresses compared to conventional fusion welding [38,39]. However, welding processes involve complex thermal and mechanical interactions that govern microstructural transformations, residual stress formation, and ultimately fatigue performance [40]. Reliable predictive models, validated against experimental data, are thus required to support design and process optimization [41,42,43]. At the same time, surface preparation and post-processing steps, such as deburring, significantly influence joint quality and must be evaluated with equal rigor [44]. The papers in this Special Issue advance this line of work by combining experimental characterization with robust numerical models that capture the coupled physics of welding and surface finishing. Mani et al. [45] use finite element modeling to analyze the thermomechanical and microstructural behavior of rotary friction welding in Inconel 718 tubes. Relevant parameters such as temperature, strain, recrystallization fraction, and grain size were predicted and validated against experimental data, showing strong agreement. The Johnson-Avrami model was applied to link thermal-mechanical conditions with microstructural evolution, accurately capturing recrystallization zone thickness and grain size variations. The findings demonstrate the effectiveness of simulation in predicting weld characteristics while reducing experimental costs. Uhlenberg et al. [46] investigate the fatigue performance of rotary friction-welded steel shafts and develop an assessment method that accounts for residual stresses. Tests under tension/compression and torsion showed that compressive residual stresses near the weld improve fatigue strength compared to fusion welding. Characterization included microstructural analysis, hardness, and residual stress measurements, supported by numerical simulations of stress formation. The fatigue assessment method proposed by the authors accurately reflected the experimental results, demonstrating its suitability as a tool for designing friction-welded components.

**Joining in Functional and Bio-Related Applications**

Although most joining research has traditionally focused on structural applications, significant challenges also emerge in functional, electronic, and biomedical fields. In this scope, the goals involve ensuring the mechanical integrity of delicate microstructures, and providing both chemical stability and durability [47]. In electronics, for example, thermal stresses during rework or assembly can compromise brittle ceramic components [48], while in biomedical systems, sealers must combine adequate strength with biocompatibility and controlled solubility [49,50]. These cases highlight that joining is a cross-disciplinary field, which extends beyond load-bearing structures [51,52]. The papers of this Special Issue on this regard show how advanced simulations and detailed material characterization can be applied to provide proper operation in sensitive environments. Yuile et al. [53] investigate the mechanical stresses that develop in fragile BaTiO_3_ multilayer ceramic capacitors during reflow desoldering operations carried out in electronic rework. A coupled fluid dynamics-finite element simulation framework was used: Fluid dynamics predicted transient temperature fields, which were then applied in finite element modelling to calculate internal stresses. The study found that the main stress driver was the thermal expansion mismatch between the capacitor and the printed circuit board (PCB), while temperature gradients within the component itself contributed only moderately. In a biomedical context, Kharouf et al. [54] compare the mechanical and physicochemical properties of three calcium silicate-based sealers: BioRoot™ Flow (BRF), CeraSeal (CRS), and TotalFill^®^. Tests included pH, porosity, roughness, flowability, compressive strength, and wettability. All materials maintained an alkaline pH, with BRF showing higher porosity and hydrophilicity but lower compressive strength than the others. Solubility exceeded 3% for all, with CRS being the least soluble.

Together, the contributions in this Special Issue highlight the latest advances in joining research. From advances in adhesive bonding to structural optimization and reinforcement strategies that enhance joint reliability, the studies describe several experimental and modeling approaches that advance the state of the art. Welding processes are examined both experimentally and numerically, providing guidelines for fatigue strength, microstructural evolution, and surface preparation. At the same time, contributions on functional and bio-related applications extend joining science and show its growing impact on electronics and biomedical technologies. On the other hand, finite element modeling, multi-physics approaches, and analytical frameworks were combined with experimental testing to validate and refine the adopted procedures. Looking forward, several directions emerge from this Special Issue. Artificial intelligence and machine learning hold great potential for real-time process monitoring, defect detection, and accelerated fatigue life prediction. Developments in multi-scale and multi-physics simulations will further enable the prediction of long-term performance in environments involving fatigue, impact, or extreme temperatures. The emphasis on sustainability, either by recyclable adhesives, defect-tolerant reinforcement strategies, or repairable joints, are other concerns to act on. As guest editor, I express my appreciation to all authors who submitted and published their work, and I hope that this Special Issue serves as an inspiration for future collaborations between academia and industry, by promoting advances in the creation of joining technologies that are stronger, more efficient, and more sustainable, while addressing the diverse performance requirements of engineering applications in multiple industrial sectors.
